# Seasonally dependent relationship between insect herbivores and host plant density in *Jatropha nana*, a tropical perennial herb

**DOI:** 10.1242/bio.035071

**Published:** 2018-07-19

**Authors:** Ashish N. Nerlekar

**Affiliations:** Department of Biodiversity, M.E.S. Abasaheb Garware College, Pune, Maharashtra 411004, India

**Keywords:** Herbivory, Resource concentration hypothesis, Resource dilution, *Jatropha nana*, Pune

## Abstract

The fact that plant spatial aggregation patterns shape insect-herbivore communities in a variety of ways has resulted in a large body of literature on the subject. The landmark resource concentration hypothesis predicts that density of insect herbivores per plant will increase as host plant density increases. I examined this prediction across temporal samplings using *Jatropha nana* and the associated specialist insect herbivores as a system. Through 12 field samplings, I modelled the effect of host plant density on insect-herbivore loads. The initial samplings (2–3) provided evidence for the resource concentration hypothesis, with insect loads increasing with increasing host plant density, whereas the later samplings (4–5, 7–11) showed the opposite; a resource dilution pattern with a decline of insect loads with increasing host plant density. These patterns also depend on the biology of the herbivores and have important implications on *J. nana* population dynamics.

This article has an associated First Person interview with the first author of the paper.

## Abstract (precis)

**Summary:** This study tests the relationship between insect-herbivore loads and host plant density across multiple time intervals and provides both evidence for and against the predictions of the landmark resource concentration hypothesis.

## INTRODUCTION

Insect herbivory is one of the most important biotic drivers that maintains the structure and function of tropical plant communities (Weissflog et al., 2018). Herbivory shapes plant community diversity, species distributions, and phenology in a variety of ways, for example through conspecific negative density dependence and altered leaf flushing patterns (Janzen, 1970; Connell, 1971; Aide, 1992). Conversely, host plant characteristics also play an important role in driving patterns of insect-herbivore abundance and diversity, through a variety of mechanisms including chemical and physical defences, nutritional content of leaves, as well as spatial and temporal variation in resources (Neves et al., 2014). Host plants and insect herbivores have been widely studied given the ecological importance of their relationships in understanding trophic interactions (Koricheva et al., 2000) and the economic importance of their relationships in crop production (Bukovinszky et al., 2005).

Out of the several hypotheses that seek to explain the relation between host plant heterogeneity, spatial complexity and insect-herbivore characteristics, three key hypotheses are the ‘enemies hypothesis’, the ‘resource concentration hypothesis’, and the ‘resource dilution hypothesis’ (Elton, 1958; Root, 1973; Otway et al., 2005; Björkman et al., 2010). The ‘enemies hypothesis’ predicts that because of higher predator and parasite efficiency in diverse environments, insect herbivores are less abundant in species-diverse plant communities than in simple (e.g. monoculture) communities (Elton, 1958). The ‘resource concentration hypothesis’ (RCH) goes further to consider host patch size and plant density as predictors of herbivore abundance, along with plant diversity (Root, 1973). Specifically, the RCH predicts that as the density of host plants or patch size increases, the density of specialist insect herbivores per plant will also increase because of the lower emigration rates from larger host patches, as these insects are more likely to find and stay in larger host patches than smaller ones. This landmark hypothesis initiated empirical testing on several systems across the world, with equivocal results (Rhainds and English-Loeb, 2003). A major development was made in this domain when Hambäck and Englund (2005), through their ‘movement based hypothesis’, provided theoretical models that could explain a much wider spectrum of patterns based on the local growth rates and migration of the insects. Through their models, they stressed that RCH is just one special case of the several possible relationships between host density and herbivore load on plants. Related to, but contrasting with the RCH, the ‘resource dilution hypothesis’ (RDH) predicts that insect-herbivore loads on host plants will instead decrease as host abundance increases (Otway et al., 2005). Resource dilution may thus reduce herbivore loads mathematically, through increased plant density and patch size relative to insect population size (Otway et al., 2005).

Along with theories investigating plant spatial structure and heterogeneity, the tremendous variation in plant investment in defences against herbivores led to the formulation of two key hypotheses: the ‘apparency theory’ and the ‘resource availability hypothesis’ (Endara and Coley, 2011). The ‘apparency theory’ of Feeny (1976) predicts that apparent perennial species, which are common and easily found by both generalist and specialist herbivores, will invest in high concentrations of chemical defences that reduce plant palatability (Feeny, 1976). On the other hand, annual unapparent species, which are difficult for herbivores to locate, invest in small quantities of highly toxic chemicals that offer protection against generalist herbivores (Feeny, 1976). The ‘resource availability hypothesis’ by Coley (1987) further predicts that slow growing species (often found in environments that constrain plant growth) are better defended compared to fast growing plants in highly productive environments. Essentially, for slow growing plants the cost of herbivory is very high, which incentivizes investment in herbivore defence. In contrast, fast growing plants with short-lived leaves can quickly replace lost tissue at less cost than energetically expensive defences (Coley, 1987).

*Jatropha nana* (Euphorbiaceae) is an endemic threatened perennial herb (referred to in literature as a dwarf under-shrub) found in fragmented populations in the states of Maharashtra, West-Bengal, Jharkhand and Bihar in India (Nerlekar et al., 2016). This species grows in distinct spatial aggregations in its natural habitat, which provide a good range of host-plant density. *J. nana* is a perennial geophyte and its shoots sprout from the tuberous rhizome in May, just before the Indian monsoon season. The aboveground tissue then wilts away by September and it remains dormant for the rest of the year through the underground rhizome, making it functionally equivalent to a herbaceous woody perennial (Nerlekar, 2015). Thus, it can be classified as an ‘unapparent’ and ‘fast growing’ species (Feeny, 1976; Coley, 1987) and we can predict that it is difficult for insects to develop a specialism for the plant, and that it also has poor defences against herbivores. However, contradictory to this prediction, Euphorbiaceae members in the genus *Jatropha* attract only a selective set of insect herbivores, owing to the secondary metabolites produced by the plants that are toxic to most insects (Shanker and Dhyani, 2006). Since the RCH is based primarily on specialist insect herbivores, I ascertained the feeding preferences of major herbivores on *J. nana* through the literature (Shanker and Dhyani, 2006; Kulkarni et al., 2009; Robinson et al., 2010) as well as pilot observations in 2014 that confirmed that this plant supported specialist insect herbivores in the study area, further making it a suitable system to test this hypothesis.

In spite of the vast body of literature available on insect herbivores and host-plant density, there are some gaps in our understanding. Most studies testing the predictions of RCH have experimented with cultivated plants and their pests as the focal species (Rhainds and English-Loeb, 2003; Tooker and Frank, 2012). Hence, our knowledge of plant–insect-herbivore relationships in wild systems is very limited in general regarding the tropics (but see Hambäck et al., 2000; Otway et al., 2005) and specifically for India (but see Thorat et al., 2016). Furthermore, the plant–insect-herbivore relationship may vary across seasons and time (Reznik, 1993), implying the need to test RCH across seasons or for the complete life span of the focal plant. In light of these gaps in our knowledge, in the present work I aimed to empirically examine the effect of the density of a tropical shrub *J. nana* on the insect-herbivore’s load in its natural habitat. For this, I used a wild host plant (*J. nana*) and the associated insect herbivores as a study system and employed a temporal field sampling approach to test the predictions of the RCH at different stages of the host plant’s life cycle.

## RESULTS

### Insect-herbivore community

Through the current sampling, I recorded a total of 17 insect-herbivore taxa (Table 1), of which five were Lepidopterans, six were Hemipterans and two were Coleopterans. I omitted four out of these 17 insects from the analysis since they were singletons. The insect community recorded showed an exceptionally high dominance of the moth *Pempelia* cf. *morosalis* (Lepidoptera: Pyralidae) (95.24% relative abundance).

**Table 1.**
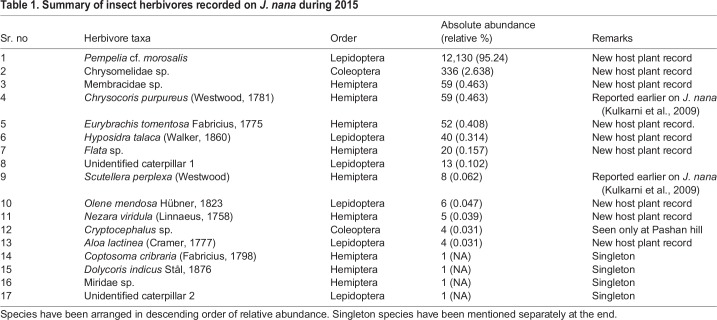
**Summary of insect herbivores recorded on *J. nana* during 2015**

### Temporal patterns of host plant density and herbivore abundance

**Fig. 1.**
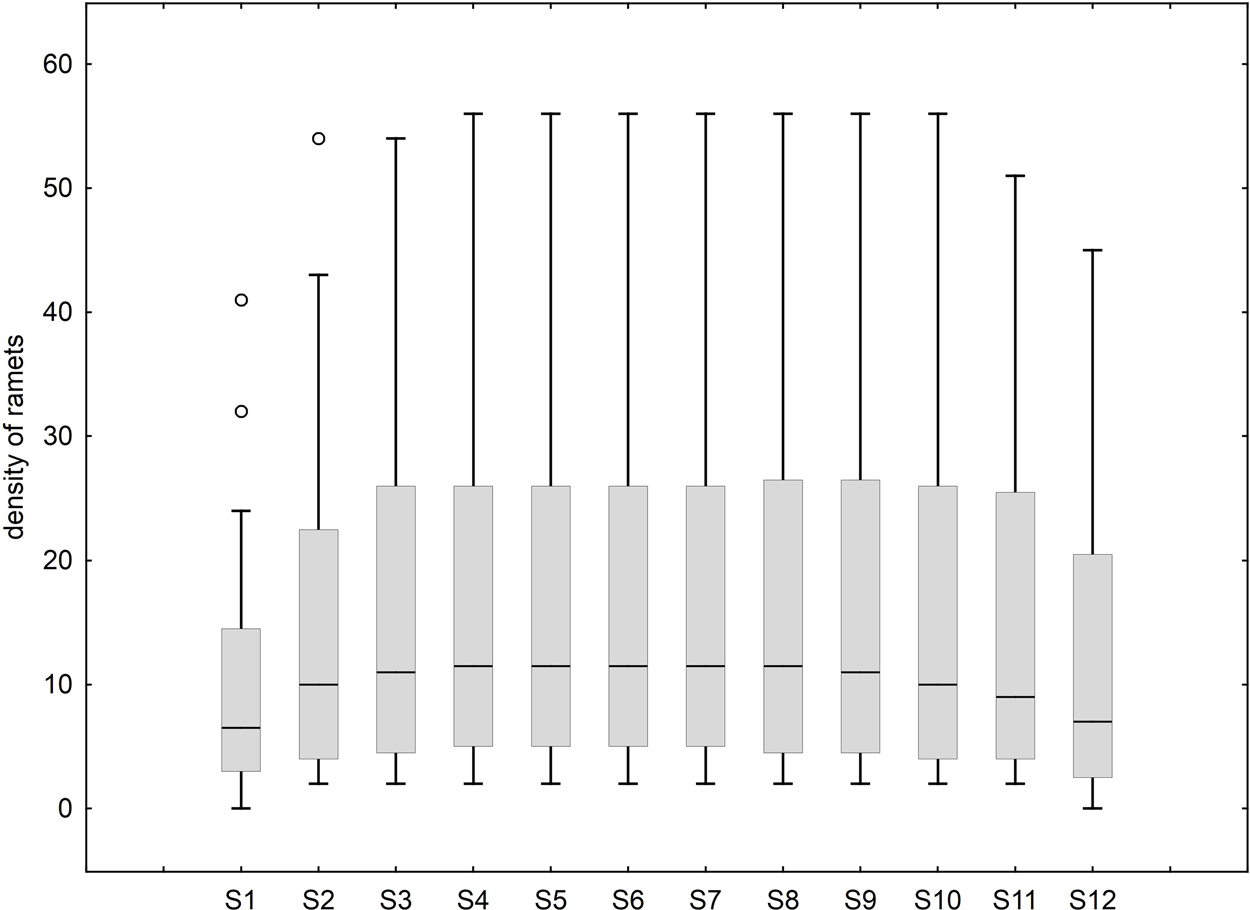
**Trend of density of host-plant ramets over samplings.** (*n*=36 clumps for each sampling). Boxes represent the inter-quartile range, the central line is the median. Whiskers represent data points less than, or, equal to upper hinge +1.5 times the inter-quartile range.

During S1, the lowest median density of ramets (6.5±9.87 SD) across *n*=36 clumps was recorded, and during S4–S8, the highest median density (11.5±14.71–14.91 SD) was recorded. The median density of ramets for all clumps increased from S1–S4 and remained constant till S9, after which it declined from S9 through S12 (Fig. 1). The median density of all the clumps was not significantly different across the 12 samplings (Kruskal-Wallis test, *H*=13.55, *P*>0.05).

**Fig. 2.**
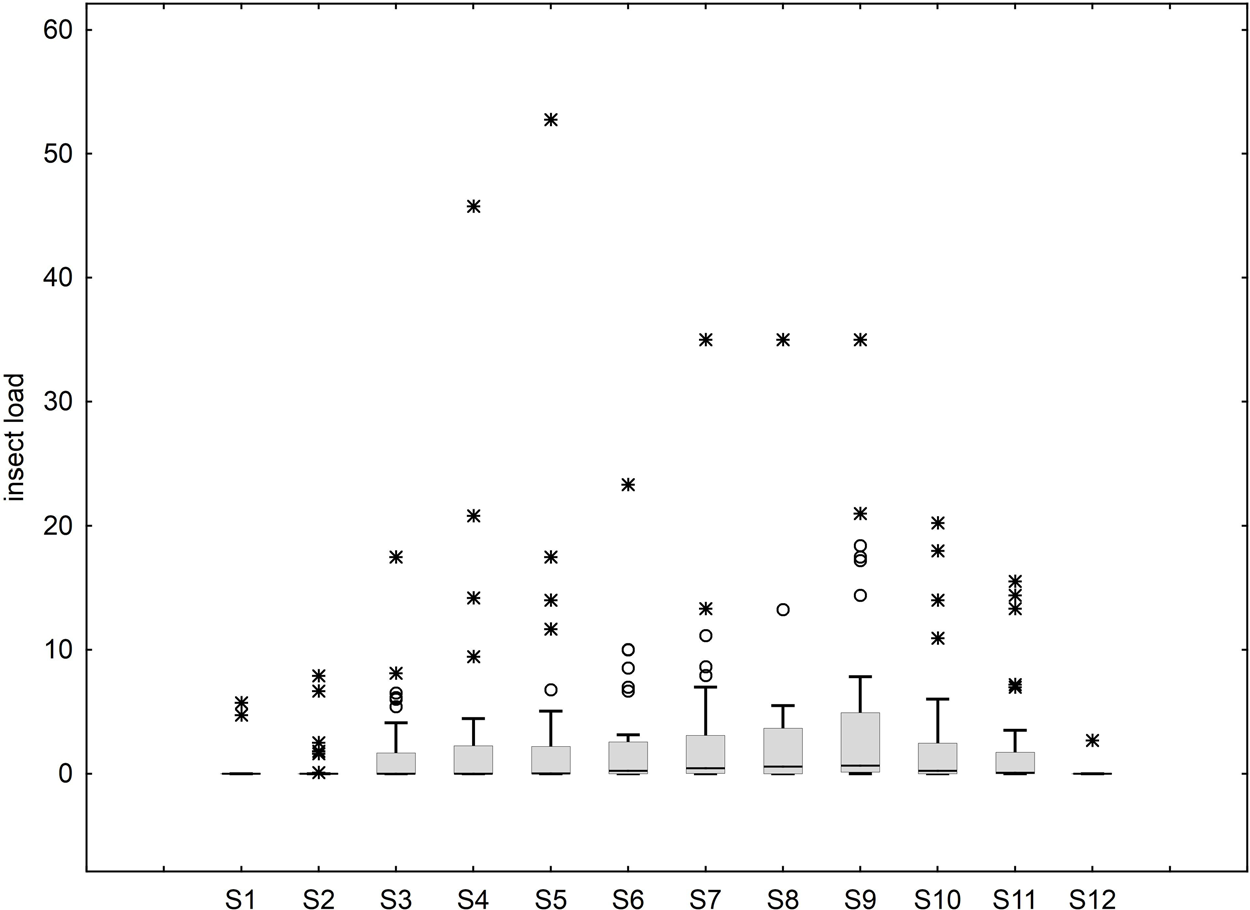
**Trend of insect-herbivore loads over samplings.** (*n*=36 clumps for each sampling). Boxes represent the inter-quartile range, the central line is the median. Whiskers represent data points less than, or equal to, upper hinge +1.5 times the inter-quartile range.

The median total abundance of insect herbivores for all clumps ranged from 0 (±11.66–75.31 SD) for S1–S4 and S12, to 9.5 (±73.29 SD) for S9 and was significantly different across samplings (Kruskal-Wallis test, *H*=81.26, *P*<0.001). Similarly, median total insect load for all clumps varied from 0 (±1.21–8.50 SD) for S1–S4, rising up to the highest of 0.630 (±7.98 SD) for S9 and then declining back to 0 (±0.44 SD) till S12 (Fig. 2). The median insect load for all the clumps together was significantly different across samplings (Kruskal-Wallis test, *H*=85.2, *P*<0.001). The pooled insect load for a seasonal analysis (seasons delimited under ‘field sampling’ section of the Materials and Methods section) revealed that there was a significant difference in median insect loads across seasons (Kruskal-Wallis test, *H*=63.02, *P*<0.001).

### Relationship between host-plant density and insect loads

**Fig. 3.**
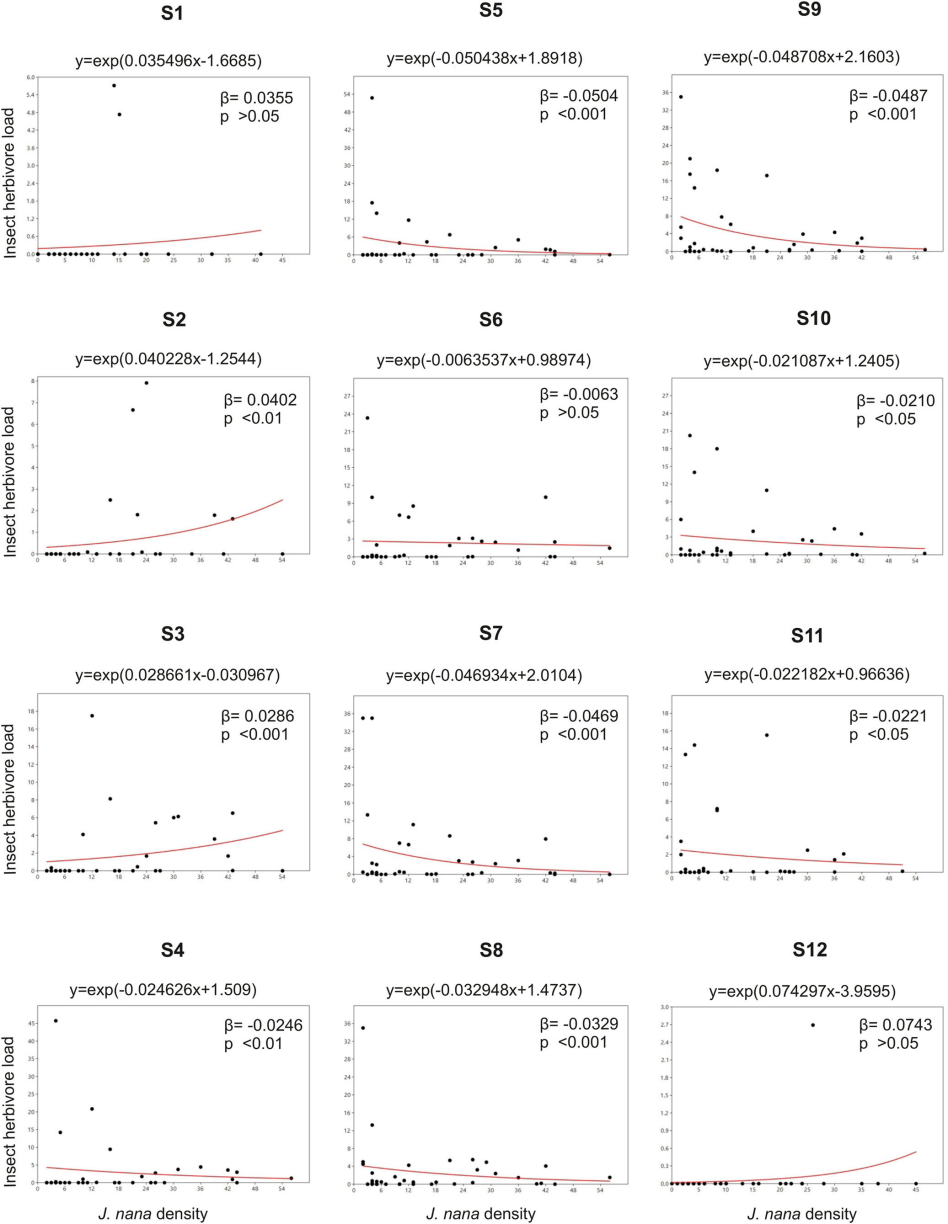
**Poisson regression models (red line) fitted to the host plant density versus insect load data for each sampling.** Samplings S2–S3 show a resource concentration pattern, whereas the samplings S4–S5 and S7–S11 show a resource dilution pattern. The model coefficient estimates (*β*) and *P* value have been provided for each sampling.

The generalized linear mixed model (GLMM) analysis for all the samplings together revealed that plant density was a significant predictor in a positive direction (*t*=8.0156; *P*<0.001) of insect loads irrespective of the temporal samplings. However, for the individual samplings overall, the results of the Poisson regression between plant density and insect loads varied across samplings. For the regressions of individual samplings, the estimates of the coefficient ranged from −0.0504 (SE=0.0094) for S5 to 0.0734 (SE=0.0418) for S12. A significant positive relationship was observed between host plant density and insect load from S2 through S3, whereas a significant negative relationship was observed from S4 through S5 and S7 through S11 (Table 2). The *G* statistic, which is the difference between the deviance of the model and another GLMM with only intercept fitted (Hammer et al., 2001), shows the highest values for S5, S7, S9, indicating the highest difference from the null (intercept) model for these samplings. Because there was a predicted significant increase in insect load per unit of plant density, it can be concluded that there was a resource concentration pattern observed for S2 and S3. Similarly for each sampling, since the insect load decreases significantly with per unit rise of host-plant density, a resource dilution effect was observed for S4 through S11 (Fig. 3).

**Table 2.**
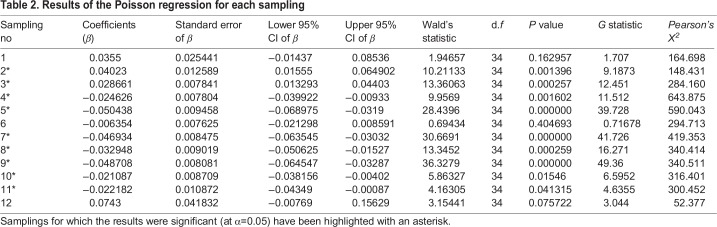
**Results of the Poisson regression for each sampling**

## DISCUSSION

The present work thus provides only partial support for the RCH and the results support the predictions of the RCH for the initial stages of the host plant. They also provide evidence for the opposite pattern of a resource dilution effect in the later stages. Samplings S2–S3 showed a positive relationship between host-plant density and insect-herbivore load. During this stage, the clumps have not reached the full density of ramets and are at a relatively initial phenology stage. A resource concentration pattern at these stages might most probably be a result of the higher emigration rates from smaller clumps and higher immigration rates into larger clumps (Root, 1973). These migration rates are directly dependent on the search mode of the insects (Bukovinszky et al., 2005) which is largely olfaction (for moths) in the present work. Through their simulated study for insects using olfaction as a search mode, Bukovinszky et al. (2005) predicted that insect loads will increase in patches of intermediate densities. The present results however show a mixed pattern, where the samplings with the highest host-plant density (S4–S5, S7–S8) show a resource dilution pattern, the ones with the lowest density (S1, S12) do not show any significant pattern, and the rest samplings (S2–S3 and S9–S11) with intermediate densities show both patterns. Considering the fact that insects can use a combination of search modes and that we know much less about this, our inferences are limited in some cases (Rhainds and English-Loeb, 2003). From S4 onwards, the insect load showed an inverse relationship such that it decreases as the host-plant density increases. Samplings S4–S10 represent the peak monsoon season and the density of ramets has reached a peak during this time. The mechanistic explanation provided by Otway et al. (2005) for such a dilution effect focusses on the inability of colonization and post colonization population growth to give rise to resource concentration.

Just as host plant densities predict insect-herbivore abundance, insect herbivory also affects the population dynamics of the host plant in different ways. Depending upon the different insect aggregation patterns, Stephens and Myers (2012) predict different outcomes for host plant population dynamics. For a resource concentration pattern, as seen for S2–S3 in this study, they predict a decline of larger patches due to higher herbivore pressure and growth of smaller patches. Similarly, for a resource dilution pattern, as seen for S4–S5 and S7–S11 in this study, larger patches are predicted to show growth and smaller ones to decline. Thus, both these insect aggregation patterns have contrasting effects on the plant population. The model by Stephens and Myers (2012) only deals with insect loads and there exists a paucity of knowledge regarding how parasites and parasitoides (on the insect herbivores) in turn affect the host plant population dynamics for various insect aggregation patterns. For the present system, given the higher abundance of these parasitoides (*Trissolcus jatrophae*, *Cheilomenes* sp., and *Telenomus* sp.) in the intermediate samplings (A.N.N, unpublished, also see Nerlekar and Rajmohana, 2016), it would be useful to model their impact on *J. nana* population dynamics. Thus, further studies that investigate patch extinction dynamics and parasitoid loads and link it with anthropogenic pressures will be a useful extension of the present work. Understanding population dynamics of *J. nana* is crucial since this species is threatened globally, and has an IUCN Red list status of ‘vulnerable’ (Nerlekar et al., 2016).

Both abiotic and biotic factors are known to shape plant phenology patterns and within biotic factors, insect herbivory has been reported to be an important selective force for phenology shifts, especially in the tropics (Aide, 1988). Leaf flushing of trees in the dry season has been proven to be a strategy to avoid peak insect herbivory in tropical seasonal forests (Aide, 1988, 1992; Murali and Sukumar, 1993). The phenology patterns of *J. nana* also show such dry season leaf and flower production, which starts in May (A.N.N, unpublished). The present data suggests that insect load is relatively lower in the pre-monsoon samplings (S1–S3) than the monsoon samplings (S4–S10). Given these phenology and insect load patterns, it would be reasonable to extend this hypothesis to this functionally woody perennial herb that also employs a dry season leaf flushing strategy to avoid higher insect herbivory in the wet season. However, higher insect load may or may not always translate into higher herbivory pressure (Rhainds and English-Loeb, 2003) (as is the case with *P. morosalis*) and directly measuring damage due to herbivory as a response across different phenology stages might be helpful in fully testing this hypothesis.

One of the limitations of this study was that the observations were only conducted for a specific time period during the day, which might have resulted in the omission of nocturnal herbivores. The RCH also hypothesises a relationship between host-patch diversity and insect loads and this was not considered in the present study. Distance between patches plays an important role in migration rates (Coley, 1987) which could not be accounted for in this work, owing to the restricted distribution of the species. Lastly, for insects I used an observation based method instead of commonly used insect traps which might have introduced bias.

## MATERIALS AND METHODS

### Study area

The hills in Pune city harbour the largest known population of *J. nana* in India (Nerlekar et al., 2016). The present study was carried out in the Vetal hills (18°31′31.04″N; 73°49′11.17″E), the Pashan-Baner hills (18°32′56.22″N; 73°47′9.16″E) and the NDA hills (18°30′1.27″N; 73°46′50.25″E) in Pune. These hills are an important natural landscape within an urban area and the habitat is a mosaic of plantations of exotic species and patches of remnant native savannah vegetation (Nerlekar and Kulkarni, 2015; https://thewire.in/environment/open-savannahs-versus-wooded-thickets-whats-the-future-for-punes-hills). On the Vetal and Pashan-Baner hills, the native *Anogeissus-Lannea-Boswellia* tree community is commonly found along with plantations of *Gliricidia sepium* (Jacq.) Kunth (Leguminosae) and *Leucaena leucocephala* (Lam.) de Wit. (Leguminosae) (Nerlekar and Kulkarni, 2015), whereas the NDA hills have fairly intact vegetation with low anthropogenic disturbance (Vattakaven et al., 2016). The elevation of the hills is about 700 m above sea level and the region receives annual seasonal rainfall (about 700 mm), of which about 90% is received between June and October. The average daily minimum temperature for January is about 11.4°C, whereas the average daily maximum temperature for April is 38.1°C. During the sampling duration (May to September, 2015), the maximum average daily temperature was recorded as 40.8°C on the 4th of May, whereas the minimum average daily temperature was 18.0°C on the 3rd of September and the total average daily rainfall was 584 mm (data from Indian Meteorological Department, Pune).

### Standardization of focal sampling unit

The study species *J. nana* grows in spatial aggregations and the radii of such aggregations vary. In order to delimit the clump radius, the degree of clumping of ramets (a single above ground shoot) was taken into account by the following formula: degree of clumping=density of all ramets in clump/average distance of all ramets from assumed centre. I noted readings of the degree of clumping for 16 such clumps for increasing radii at 1 m interval from the centre. Then I plotted the degree of clumping versus area of circle. The radius at which the value for degree of clumping flattens was found to be 80 m^2^ area (=5 m radius) which was used as the radius for all clumps and kept constant during all samplings (Fig. S1). Thus, the focal observation area was kept constant, within which the individual ramets’ densities were recorded.

### Field sampling

Through pilot ground surveys in 2014, I traced and mapped clumps of *J. nana* in the study area. I carried out field sampling 12 times [intervals between sampling range from 5–8 days (average 6.6 days) for sampling 1 and sampling 11, and 16 days between sampling 11 and sampling 12 (see Table S1 for sampling dates)] from May to September 2015 (referred to as S1–S12 hereon). In each sampling effort I measured 36 stratified random clumps, proportional to the total estimated population on each of the hills (23 on Vetal hills, eight on NDA hills and five on Pashan hills) based on varying initial densities. I divided a typical sampling into three to four days (an average of four hours/day) and sampled within a fixed time range of 1300 h to 1700 h. In each sampling I noted the density of ramets and presence of insect herbivores in each clump. The sampling period covers a seasonal transition from late summer (S1–S2, May, hot and dry with intermittent thunderstorms, most monsoon herbs in dormant stage, *J. nana* ramets sprouting from tubers) to early monsoon (S3–S5, June, intermediate rainfall, monsoon herbaceous vegetation in initial growth stages, *J. nana* ramets flowering), mid monsoon (S6–S10, June–August, maximum rainfall, peak flowering, fruiting for most monsoon herbaceous vegetation including *J. nana*) and late monsoon (S11–S12, August–September, intermediate rainfall with minimum mean minimum daily temperature, monsoon herbaceous vegetation about to dry, most *J. nana* ramets wilted and fruits fully mature) for the study area.

On all the ramets within the clump, I scanned insect herbivores visually by turning at least 50 % of randomly selected leaves by hand to check the underside, and recorded the insect species along with their abundance. For *Pempelia* cf. *morosalis* caterpillars, I recorded the abundance as follows: due to the webbing behaviour of the caterpillars, it was difficult to estimate absolute abundance without employing invasive methods. Hence, I scored the damage due to consumption as low, moderate or high. The low damage stage had about 70 individuals, 40 individuals for moderate and ten individuals for high, as found out by pilot observations (The low damage stage has more individuals than the high damage stage). I ascertained an insect as herbivore only if it was seen damaging any plant part in the field or the lab, or if it was indicated in the literature. Thus, I omitted insects that were currently at life stages that do not feed on plants (for example eggs, pupae) from the analysis. I also excluded herbivores that occurred only once in all the samplings combined (singletons) from the analyses. I collected insect specimens, preserved in 70 % ethanol, which were identified using standard keys (Hampson, 1892–1896; Distant, 1977) and validated by experts. It was not possible to identify some insects at the species level due to multiple factors including lack of adequate number of specimens, lack of specimens at the adult stage (some moths were only recorded as larva and could not be successfully reared in the lab for recording adult morphology) and finally lack of revised and updated literature. In all such cases, I identified the insects to the lowest taxonomic level possible.

### Data analysis

All statistical analysis was performed in PAST (Hammer et al., 2001) and STATISTICA (StatSoft, 2001). I checked data for normality by using the Shapiro-Wilk's test and found it to be non-normal, therefore I chose the non-parametric Kruskal-Wallis test to check if there was a significant difference between sampling for density of ramets and insect loads. Insect load has been defined as total abundance of all insects divided by the total number of ramets in the clump (Otway et al., 2005). For all the tests, results were considered significant at the α =0.05 threshold level. To check if the insect-herbivore load was significantly predicted by plant density for all samplings combined, I used GLMM with Poisson distribution and log link function, keeping sampling numbers as a random factor. Similarly, to model the impact of plant density on insect-herbivore loads for each sampling separately, I compared model coefficients using a Poisson regression (with clump numbers as a random factor) within the GLMM framework, with log link owing to the Poisson distribution of data (Agresti, 2007).

## Supplementary Material

Supplementary information

First Person interview
