# Supplementary material for: Seasonally dependent relationship between insect herbivores and host plant density in Jatropha nana, a tropical perennial herb
Source: Biol Open. 2018 Jul 19;7(8):bio035071. doi: 10.1242/bio.035071 (PMC6124573; doi:10.1242/bio.035071)
Supplement: Supplementary information [file biolopen-7-035071-s1.pdf]

Table S1: Sampling dates with climatic data for the respective dates. Climatic data obtained from Indian Meteorological Department, Pune.

**Sampling no.**	**Sampling dates**	**Average of daily minimum temperature (°C)**	**Average of daily maximum temperature (°C)**	**Average of daily precipitation (mm)**
S1	17 to 20th May 2015	23.8	37.0	0.0
S2	28 to 31st May 2015	23.3	36.4	0.3
S3	8 to 11th June 2015	22.3	34.1	7.9
S4	17 to 20th June 2015	22.2	29.5	1.8
S5	28th June to 1st July 2015	21.9	30.4	0.5
S6	7 to 10th July 2015	23.3	30.2	0.8
S7	18 to 21st July 2015	22.6	27.6	4.0
S8	28 to 31st July 2015	21.5	27.9	4.4
S9	8 to 10th August 2015	21.7	28.9	0.4
S10	18 to 20th August 2015	20.9	31.2	0.0
S11	29 to 31st August 2015	21.2	29.9	3.0
S12	17 to 19th September 2015	22.7	28.4	16.8

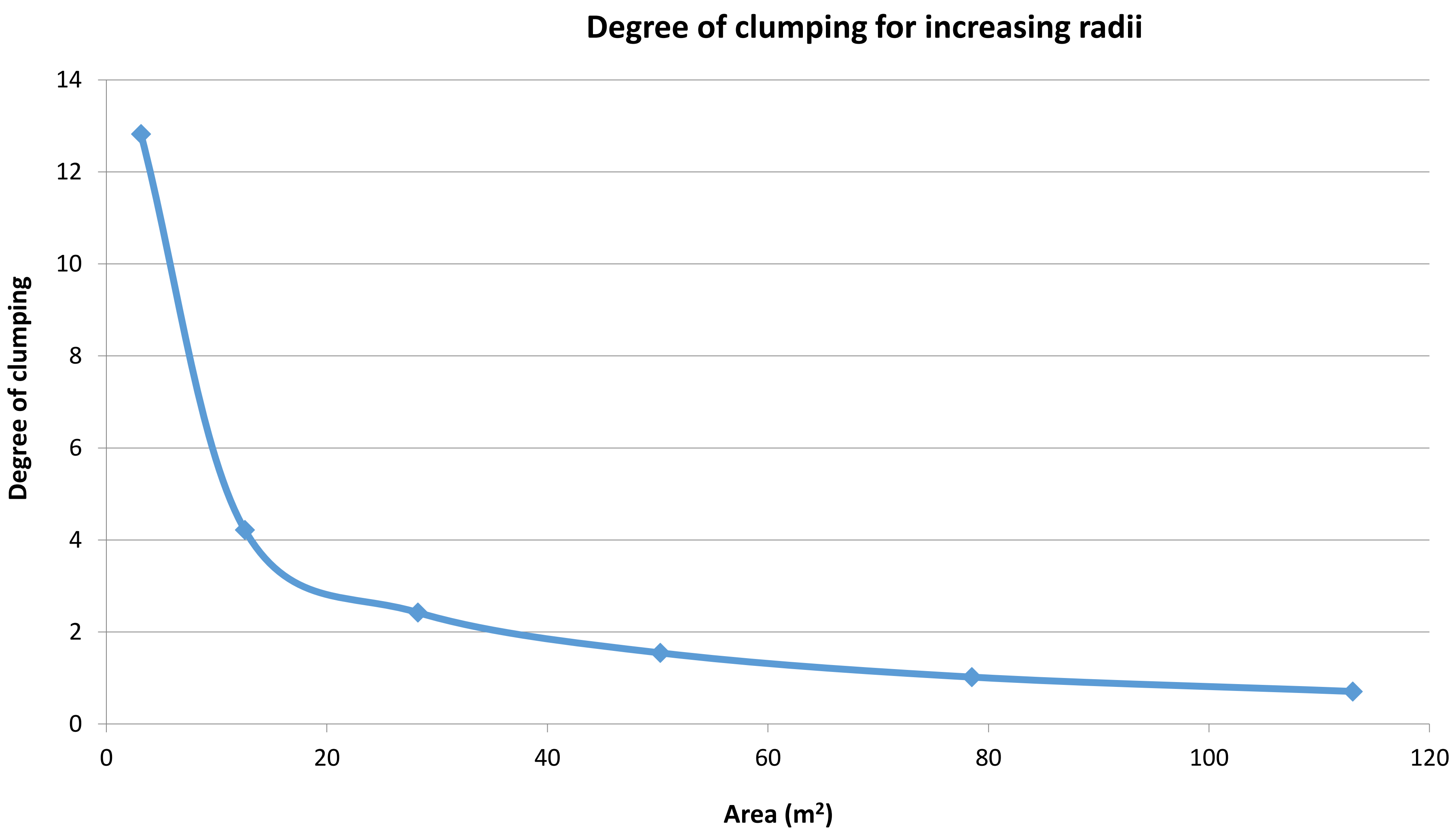

Figure S1. Degree of clumping with respect to increasing area (n=16 clumps).
