# Supplementary material for: Seasonally dependent relationship between insect herbivores and host plant density in Jatropha nana, a tropical perennial herb
Source: Biol Open. 2018 Jul 19;7(8):bio035071. doi: 10.1242/bio.035071 (PMC6124573; doi:10.1242/bio.035071)
Supplement: First Person interview [file biolopen-7-035071-s2.pdf]

FIRST PERSON

# First person – Ashish Nerlekar

First Person is a series of interviews with the first authors of a selection of papers published in Biology Open, helping early-career researchers promote themselves alongside their papers. Ashish Nerlekar is first author on 'Seasonally dependent relationship between insect herbivores and host plant density in *Jatropha nana*, a tropical perennial herb', published in BiO. Ashish's research focuses on plant community ecology, specifically savanna ecology, urban biodiversity and taxonomy, most recently within the urban savannas of Pune, India.

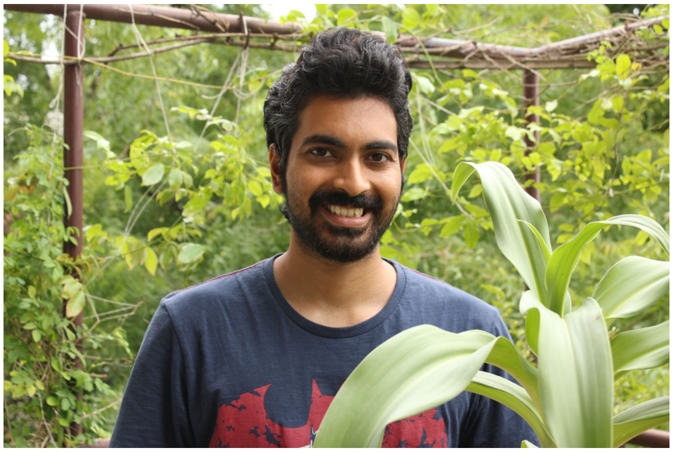

Ashish Nerlekar

**What is your scientific background and the general focus of your lab?**

Having completing my bachelor's degree in botany and master's degree in biodiversity, I have acquired a broad training in natural history, plant taxonomy and ecology. As a part of my master's project, I worked on the taxonomy, conservation and ecology of a plant, *Jatropha nana*, found in and around the urban green-scapes of the city of Pune, India. Since then, I have worked on projects dealing with the restoration of grasslands, molecular phylogeny of a plant group endemic to Western Ghats, and recently, on a project that investigates thermotolerance of tropical herbs belonging to different functional types. These opportunities helped me focus on savanna plant community ecology for further research and I have been working on the urban savannas of Pune for the last few years. Through my research on urban savannas, I aim to ultimately generate strong citizen support to save these unique and fast-deteriorating habitats.

**How would you explain the main findings of your paper to non-scientific family and friends?**

Some insects are very choosy and need only one particular type of plant to complete their life cycle. One such plant on which some insects exclusively depend is the dwarf jatropha. For my research, I asked a very simple question: do more jatropha plants result in more insects (per plant) that feed on them? My data says both yes and no, and that it depends on several factors like seasonality. Interestingly, I found that the 'more plants means more insects per plant' prediction is true only in the initial growth season of the plant. For the longer remaining season, an opposite pattern is seen – more plants in fact mean fewer insects (per plant) feeding on them. The dwarf jatropha is found only in a few places and in small numbers and hence is at risk of extinction. Insects that feed on this plant also alter its population. This is why studying insect–plant relationships is very important.

**What are the potential implications of these results for your field of research?**

In spite of the recent theoretical models proposed for understanding the relationship between host plant density and insect herbivore loads, empirical evidence for these models from tropical wild systems is currently limited. This work highlights patterns for one such wild dwarf herb in a seasonally dry urban savanna. I hope that similar research will be initiated on tropical wild plants belonging to various functional groups and in different biogeographic zones to validate the proposed models.

Ashish Nerlekar's e-mail: ashishnerlekar3@gmail.com

**How did this research help advance your career?**

Since this was my first independent venture, I learnt a lot across the entire process. This was also my first successful attempt at generating independent funding for my research. The project, as a whole, helped me in several ways. Firstly, I formed my first international collaboration for developing allometric equations to predict the leaf area of this species. Secondly, through this project, I got the opportunity to network with a diverse set of researchers from insect taxonomists and herbarium curators to statisticians. It was a fantastic experience to learn from all of them. Thirdly, the several outcomes of this project in the form of scientific and popular articles and poster presentations provided much-needed training in science communication. Lastly, I came to appreciate the tremendous importance of the socio-economic factors that drive and shape the urban landscape ecology.

> "Research on Indian savannas has recently come into the limelight, and this will surely make a difference in how managers and citizens perceive the value of such landscapes."

**What, in your opinion, are some of the greatest achievements in your field and how has this influenced your research?**

On a global scale, I believe the recent research on expanding the 'old growth' concept to savannas has been a major breakthrough in the domain of savanna ecology. This perspective and allied research provides an objective framework for ecologists to distinguish between primary versus secondary and derived savannas across different biogeographic zones. The steadily increasing research on antiquity of global savannas and drylands using multi-proxy approaches, coupled with the appreciation of the roles of anthropogenic drivers has been another positive development in this field.

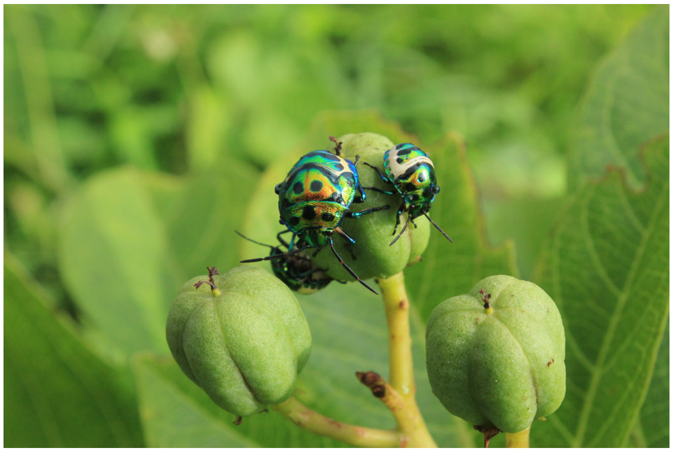

Instars of the bug *Chrysocoris purpureus* feeding on the fruits of *Jatropha nana*.

Research on Indian savannas has recently come into the limelight, and this will surely make a difference in how managers and citizens perceive the value of such (earlier considered ‘barren’) landscapes. Seminal papers on this theme have changed the way ecologists classify forested landscapes, with the old classification based on timber trees replaced by a classification based on functional traits of Indian savannas. It is because of these recent advances that I could place my research on the ecology and conservation of *Jatropha nana* in the broader context of an urban savanna.

### What changes do you think could improve the professional lives of early-career scientists?

First-hand exposure to cutting-edge science, tools and scientific practices, constructive reviews on manuscripts and better funding for pure sciences.

### What’s next for you?

I will be starting my PhD on savanna ecology in the fall of 2018 with Dr Joseph Veldman at Texas A&M University, USA. I hope to better understand the structural and functional characteristics of ‘old-growth’ and secondary savannas as a part of my doctoral work.

#### Reference

**Nerlekar, A. N.** (2018). Seasonally dependent relationship between insect herbivores and host plant density in *Jatropha nana*, a tropical perennial herb. *Biol. Open* **7**: bio035071.
